# Type IIb Heat Labile Enterotoxin B Subunit as a Mucosal Adjuvant to Enhance Protective Immunity against H5N1 Avian Influenza Viruses

**DOI:** 10.3390/vaccines8040710

**Published:** 2020-11-30

**Authors:** Neos Tang, Chun-Yi Lu, Shih-Che Sue, Ting-Hsuan Chen, Jia-Tsrong Jan, Ming-Hsi Huang, Chung-Hsiung Huang, Chung-Chu Chen, Bor-Luen Chiang, Li-Min Huang, Suh-Chin Wu

**Affiliations:** 1 Institute of Biotechnology, National Tsing Hua University, Hsinchu 30013, Taiwan; tomtang1107@gmail.com (N.T.); sadam1114@gmail.com (T.-H.C.); 2 Department of Pediatrics, National Taiwan University Children Hospital, Taipei 100226, Taiwan; cylu@ntu.edu.tw (C.-Y.L.); gicmbor@ntu.edu.tw (B.-L.C.); lmhuang@ntu.edu.tw (L.-M.H.); 3 Department of Life Science, Institute of Bioinformatics and Structural Biology, National Tsing Hua University, Hsinchu 30013, Taiwan; scsue@life.nthu.edu.tw; 4 Genomics Research Center, Academia Sinica, Taipei 11529, Taiwan; tsrong33@gate.sinica.edu.tw; 5 National Institute of Infectious Diseases and Vaccinology, National Health Research Institutes, Zhunan 35053, Taiwan; huangminghsi@nhri.org.tw; 6 Department of Food Science, National Taiwan Ocean University, Keelung 202301, Taiwan; huangch@mail.ntou.edu.tw; 7 Department of Internal Medicine, MacKay Memorial Hospital, Hsinchu 30013, Taiwan; 4059@mmh.org.tw; 8 Teaching Center of Natural Science, Minghsin University of Science and Technology, Hsinchu 202301, Taiwan; 9 Department of Medical Science, National Tsing Hua University, Hsinchu 30013, Taiwan

**Keywords:** intranasal immunization, mucosal adjuvant, LTIIb-B5, H5N1 vaccine

## Abstract

Human infections with highly pathogenic avian influenza H5N1 viruses persist as a major global health concern. Vaccination remains the primary protective strategy against H5N1 and other novel avian influenza virus infections. We investigated the use of *E. coli* type IIb heat labile enterotoxin B subunit (LTIIb-B5) as a mucosal adjuvant for intranasal immunizations with recombinant HA proteins against H5N1 avian influenza viruses. Use of LTIIb-B5 adjuvant elicited more potent IgG, IgA, and neutralizing antibody titers in both sera and bronchoalveolar lavage fluids, thus increasing protection against lethal virus challenges. LTIIb-B5 mucosal adjuvanticity was found to trigger stronger Th17 cellular response in spleen lymphocytes and cervical lymph nodes. Studies of anti-IL-17A monoclonal antibody depletion and IL-17A knockout mice also suggest the contribution from Th17 cellular response to anti-H5N1 protective immunity. Our results indicate a link between improved protection against H5N1 live virus challenges and increased Th17 response due to the use of LTIIb-B5 mucosal adjuvant with HA subunit proteins.

## 1. Introduction

Rapidly evolving influenza A viruses have resulted in several widespread infections, including the 1918 H1N1 (“Spanish flu”), 1957 H2N2 (“Asian flu”), 1968 H3N2 (“Hong Kong flu”), 1977 H1N1 (“Russian flu”), and 2009 H1N1 (“Mexican flu”) pandemics. The first widespread case of highly pathogenic avian influenza (HPAI) H5N1 virus infection in humans occurred in Hong Kong in 1997 and re-emerged in 2003. Since then, sporadic outbreaks of human infections have been identified in Asia, the Middle East, Europe, and Africa, with an overall mortality rate of approximately 60% [1,2,3,4]. HPAI H5N1 viruses primarily affect lower respiratory tracts, resulting in lung damage and plasma cytokine dysregulation [5,6]. Although HPAI H5N1 viruses are not efficiently transmitted between humans, at least two research teams have reported that HA protein mutations lead to effective transmission in ferret models [7,8]. Thus, there is increasing concern about the potential threat of H5N1 and other HPAI types to human populations. Vaccination remains the primary protective strategy against influenza outbreaks. There are several reasons why this strategy is considered limited for influenza pandemics, the most important being the stockpiling the correct antigen in case of a pandemic or being able to quickly produce the pandemic vaccine after the identification of the viral strain. Adjuvants may be helpful in two ways: enhancing immunogenicity, and reducing the required antigen quantity [9].

Mucosal immunization is considered a potentially useful strategy, with studies showing its ability to induce robust immune responses where most infections begin. Delivery route-nasal, sublingual, oral, rectal, vaginal or trans-dermal-is an important focus on mucosal vaccine development research because it determines the extent of protection [10]. For airway protection, nasal delivery provides a respiratory system defense that generates both antigen-specific mucosal immunity and systemic immunity against foreign antigens [11,12]. Antigens are recognized in nasopharynx-associated lymphoid tissues containing M cells, antigen-presenting cells, T cells, and B cells, thereby triggering mucosal immune responses such as IgA-secreting B and plasma cells [12]. To elicit effective mucosal immunity, nasal vaccine delivery requires the use of an adjuvant that might involve toll-like receptor (TLR) ligands [12,13,14]. For the present study we considered *E. coli* type IIb heat labile enterotoxin B subunit (LTIIb-B5) which triggers immune responses via the TLR-2/1-dependent activation of pattern recognition receptors [15,16]. The TLR-2/1-dependent activation of LTIIb-B5 is not found in the B subunits of type I heat labile enterotoxins such as the cholera toxin (CT) and *E. coli* heat labile enterotoxin (LT-I) [16]. In contrast, LTIIb-B5 stimulates dendritic cell activation and maturation, and induces CD4^+^ T cell proliferation. Furthermore, LTIIb-B5 has been described as enhancing IgA secretion in mucosal vaccination routes [15,16].

In this study, we used a mouse model to analyze the potential of LTIIb-B5 proteins for use as a mucosal adjuvant for intranasal immunizations against H5N1 infections. Mucosal immunizations in BALB/c mice with HA antigen plus LTIIb-B5 adjuvant were conducted to investigate the elicited titers of HA-specific IgG, IgA, and neutralizing antibodies in sera and bronchoalveolar lavage (BAL) mucosal fluids, and the induction of HA-specific T cell responses (Th1, Th2, Th17) in spleen lymphocytes (SPLs) and cervical lymph nodes (CLNs). Protective immunity was determined against live virus challenges. Our results indicate a link between improved protection against H5N1 live virus challenges and increased Th17 response due to the use of LTIIb-B5 mucosal adjuvant with HA proteins. These findings may provide useful information for mucosal H5N1 subunit vaccine development.

## 2. Experimental Section

### 2.1. Recombinant LTIIb-B5 Cloning, Expression, and Purification

The codon-optimized type IIb heat labile enterotoxin B subunit (LTIIb-B5) gene (accession No. P43529) was cloned into a pET22b (+) expression vector with a C-terminal 6 His-tag. LTIIb-B5-pET22b (+) plasmids were transformed into *E. coli* BL21 cells (DE3) (Invitrogen) and grown in Luria-Bertani (LB) broth. Recombinant proteins were produced 4 h following 1 mM IPTG stimulation. Cells were collected by centrifugation at 5000 rpm for 15 min at 4 °C. Pellets were resuspended in 40 mL buffer A (300 mM Tris, 50 mM NaCl, 10 mM imidazole, 5% glycerol; pH 7.2) with 1 mM PMSF (USB) for purification. Cells were homogenized at 15k PSI. Inclusion bodies were solubilized with 8M urea, eluted with 30–40% Buffer B (300 mM Tris, 50 mM NaCl, 500 mM imidazole, 5% glycerol; pH 7.2) by Nickel affinity chromatography column, and dialyzed with 1 × PBS at 4 °C overnight. Purified proteins were concentrated using 10 K centrifugal filters (MILLPORE), passed through an endotoxin removal column (Cellfine), analyzed using sodium dodecyl sulfate polyacrylamide gel electrophoresis (SDS-PAGE) with Coomassie blue staining and western blotting using anti-His horseradish peroxidase (HRP)-conjugated antibodies (Affymetrix). Dithiothreitol (DTT) was used to disrupt the disulfide bond formation in SDS-PAGE gels. Limulus amoebocyte assay kit was used to measure the residual LPS content of LTIIb-B5. Sephacryl S-100 packed in 16/60 gel filtration columns (GE) was used for protein quaternary structure and composition analyses. For an NMR sample [15], N-labeled LTIIb-B5 was prepared using an M9 minimal medium supplemented with [15] NH_4_Cl (1 g/L) as the sole nitrogen source. A sample containing approximately 0.2 mM proteins in 50 mM Tris buffer (pH 6.0) and 150 mM NaCl in 95% H_2_O/5% D_2_O was used for NMR measurements. NMR HSQC experiments were conducted at 25 °C using a Bruker AVANCE spectrometer (850 MHz).

### 2.2. Functional TLR Ligand Assays

HEK 293A cells were seeded and held overnight in culture dishes (6 × 10^6^ cells/dish) prior to co-transfection with pDUO-hTLR1/hTLR2 plasmids (InvivoGen, San Diego, CA, USA) (7.5 μg/dish) and pGL4.32 (luc2p/NF-κB-RE/Hygro) vectors (Promega) (3 μg/dish) using Turbofect (Thermo Fisher Scientific, Waltham, IL, USA) transfection reagent. Transfected cells were seeded into 96-well plates at a density of 5 × 10^4^ cells/well. The following day, recombinant LTIIb-B5 or Pam3CSK4 (InvivoGen, San Diego, CA, USA) was prepared for serial dilutions (10 μg/mL to 1 pg/mL) that were used to treat batches of cells for 5 h. Treated cells were lysed with Glo-lysis buffer (Promega, Madison, WI, USA). Luciferase activity was determined by adding neolite luciferase substrate, and observed with a Victor II microplate reader (both PerkinElmer, Waltham, IL, USA).

### 2.3. Recombinant HA Protein Expression and Purification

Recombinant HA proteins were generated as previously described [17,18]. For recombinant HA production, Sf9 cells were grown in SF900-II medium (Invitrogen, Carlsbad, CA, USA) and infected with recombinant baculoviruses at 2 × 10^6^ cells/mL for 48 h. Recombinant HA proteins were purified by nickel affinity chromatography and eluted with 50–100% Buffer B. Purified HA proteins were concentrated using 30 kDa-restricted centrifugal filters (Millipore, Burlington, VT, USA), dialyzed with PBS, and stored at −20 °C.

### 2.4. Mouse Immunization and Sample Collection

Groups of female BALB/c and C57BL/6 mice (6–8 weeks old) were purchased from National Laboratory Animal Center, Taiwan. The source of IL-17A knockout mice (backcrossed to C57BL/6J for 8 generations) was obtained from Dr. Ryo Goitsuka, Tokyo University of Science. These mice were intranasally immunized with 5, 10 or 15 μg recombinant HA plus 1 or 5 μg recombinant LTIIb-B5 proteins, most in the formulation of 10% PELC oil-in-water emulsions [18,19]. PELC is a squalene-based oil-in-water emulsion stabilized by a combination of Span^®^85 (sorbitan trioleate) (Sigma-Aldrich, St. Louis, MO, USA) and poly(ethylene glycol)-block-poly(lactide-co-ε-caprolactone) (PEG-b-PLACL) [18,19]. For intranasal immunizations, mice were anesthetized using Zoletil (Virbac, Carros, France) prior to the application of 20–30 μL solution containing recombinant HA (with or without LTIIB-B5) or a control. All mouse groups were immunized three times (at weeks 0, 3 and 6); serum samples were collected at week 8. Most mice were sacrificed at week 9 for purposes of collecting splenocytes, CLNs, and BAL fluid. All samples were stored at −20 °C until used for analyses. All procedures involving animals were performed in accordance with the guidelines established by the Laboratory Animal Center of National Tsing Hua University (NTHU). Animal use protocols were reviewed and approved by the NTHU Institutional Animal Care and Use Committee (approval no. 10246).

### 2.5. HA-Specific Antibody Titer Analysis

ELISAs were used to measure antibodies in immunized mouse sera and BAL fluid samples. After coating with 2 μg/mL of recombinant HA protein, 96-well ELISA plates (Corning Inc., Corning, NY, USA) were held overnight at 4 °C, blocked with 1% BSA in PBS at room temperature, and held for 30 min. Serially diluted samples were added to each plate and incubated for 1 h at room temperature. Next, HRP conjugated goat anti-mouse IgG antibodies (1:30,000) or HRP conjugated goat anti-mouse IgA antibodies (1:10,000) (Bethyl Laboratories, Montgomery, AL, USA) were added to individual plates and held for another 1 h at room temperature. TMB substrates (BioLegend, San Diego, CA, USA) were added for coloration, followed by incubation for 15 to 20 min at room temperature; reactions were stopped with 2 N H_2_SO_4_ and detected using an ELISA reader (OD_450_). End-point titers were measured as the 4-fold absorbance of a negative control.

### 2.6. Neutralization Assays

Neutralizing antibodies were quantified as reduced luciferase expression levels following H5N1 pseudotyped lentiviral particle (H5N1pp) transduction in MDCK cells [20,21]. H5N1 pp can only initiate a single cycle infection but with a similar entry characteristics of receptor usage, pH requirement and neutralization compared to the wildtype H5N1 viruses. Since no progeny viruses can be produced from pseudotyped lentiviral particles, the H5N1pp assay can be performed under low biosafety requirements. Briefly, HEK293T cells were co-transfected with pNL Luc E^−^ R^−^ and pcDNA3.1(+) expressing HA from A/Thailand/1(KAN-1)/2004 strain, and pcDNA4B expressing the NA of the A/Viet Nam/1203/2004 strain. Culture supernatants were collected and concentrated 48 h post-transfection. H5N1pp titer was determined by p24 ELISA (Clontech Laboratories, Mountain View, CA, USA). Next, 50 µL H5N1pp (50 TCID_50_) were incubated with 50 µL diluted antisera (two-fold serial dilution starting from 1:40) for 1 h at 37 °C, followed by the addition of MDCK cells (1.5 × 10^4^ cells/well). Cells were lysed with Glo-Lysis Buffer 2 days post-infection. Luciferase activity was measured following the addition of neolite luciferase substrate. Neutralization titers (IC50) were measured as the reciprocal of serum dilution required to obtain a 50% reduction in RLU compared to a control containing the H5N1pp virus only. Neutralization curves and IC50 values were analyzed using GraphPad Prism 5 Software.

### 2.7. Cytokine Analysis

SPL and CLN cells were seeded into 96-well plates (5 × 10^5^ cells/well) and stimulated with 1 μg/mL pooled HA peptides (15-mer overlapped by eight amino acids spanning the HA_1_ of A/Viet Nam/1203/2004/H5N1) and 5% CO_2_ for 3 days at 37 °C. Cultured supernatants were collected and stored at −20 °C. ELISA plates (96-well) were coated with IFN-γ, IL-4, IL-17A or IL-22 capture antibodies, held overnight at 4 °C, and blocked with 1% BSA according to the manufacturer’s instructions (BioLegend, San Diego, CA, USA). Diluted samples were incubated with capture antibodies for an additional 2 h at RT. Cytokines were detected using specific antibodies for 1 h each, and interacted with avidin-HRP for 30 min prior to determining coloration and end-point titers.

### 2.8. Flow Cytometry

SPL and CLN stimulation was performed as described above, with 5 μg/mL Brefeldin A (Sigma-Aldrich, St. Louis, MO, USA) added before the last 6 h of incubation. Samples were fixed with 4% formaldehyde at RT for 30 min prior to washing and blocking with 40 µL staining buffer (2% FBS + 0.01% NaN_3_ in 1× PBS). Anti-mouse CD4 antibody (1 μg/mL) conjugated with PE (BioLegend, San Diego, CA, USA) was used for CD4 staining. Samples were resuspended with permeabilization buffer (eBioscience, San Diego, CA, USA) and intracellularly stained with 1 μg/mL anti-mouse IL-17A antibody conjugated with PerCP-Cy5.5 (eBioscience, San Diego, CA, USA). Flow cytometric analyses were performed using a BD Accuri C6 flow cytometer. BD Accuri CFlow v1.0.264.21 software was used to analyze the data.

### 2.9. Virus Challenges

Groups of BALB/c mice, C57BL/6 mice, IL-17A knockout mice were intranasally immunized with three doses of recombinant HA without or with LTIIb-B5 adjuvant formulated in PELC oil-in-water emulsions or three doses of PBS as a mock control. Immunized mice were challenged intranasally with H5N1 NIBRG-14 virus (A/Vietnam/1194/2004) (H5N1) 3 weeks following the third dose immunizations at a 10-fold mouse median lethal dose. For IL-17A depletion, two groups of BALB/c mice immunized with 10 μg HA + 5 μg LTIIb-B5 + PELC were intraperitoneally injected with 0.1 mg of anti-IL-17A monoclonal antibody (mAb) (R&D SYSTEMS) or IgG2A isotype control mAb (R&D SYSTEMS) at 1 day before and 1, 3, 5 days after viral challenges. Survival rates and body weights were recorded daily for 14 days. Body weight loss >25% was used as an end-point.

### 2.10. Histopathology

One week after final vaccination, the mice were sacrificed and the lung tissues were harvested, fixed in 10% buffered formalin and embedded in paraffin. Sections of 5 μm were stained with hematoxylin and eosin (H&E) by the Pathology Core Laboratory of NHRI for histological examination using an Olympus DP70 microscope (×40 magnification).

### 2.11. Statistical Analyses

All data in this study were calculated by GraphPad Prism V6.01. Two-tailed student’s *t*-tests or one-way ANOVA analysis with Tukey’s or Holm-Sidak’s multiple comparison tests were used to analyze all results except survival data, and the Kaplan-Meier analysis methods were adopted in the survival analysis, with statistical significance determined as *p* < 0.05.

## 3. Results

### 3.1. Expression, Purification, and Characterization of Recombinant LTIIb-B5 Proteins

**Figure 1:**
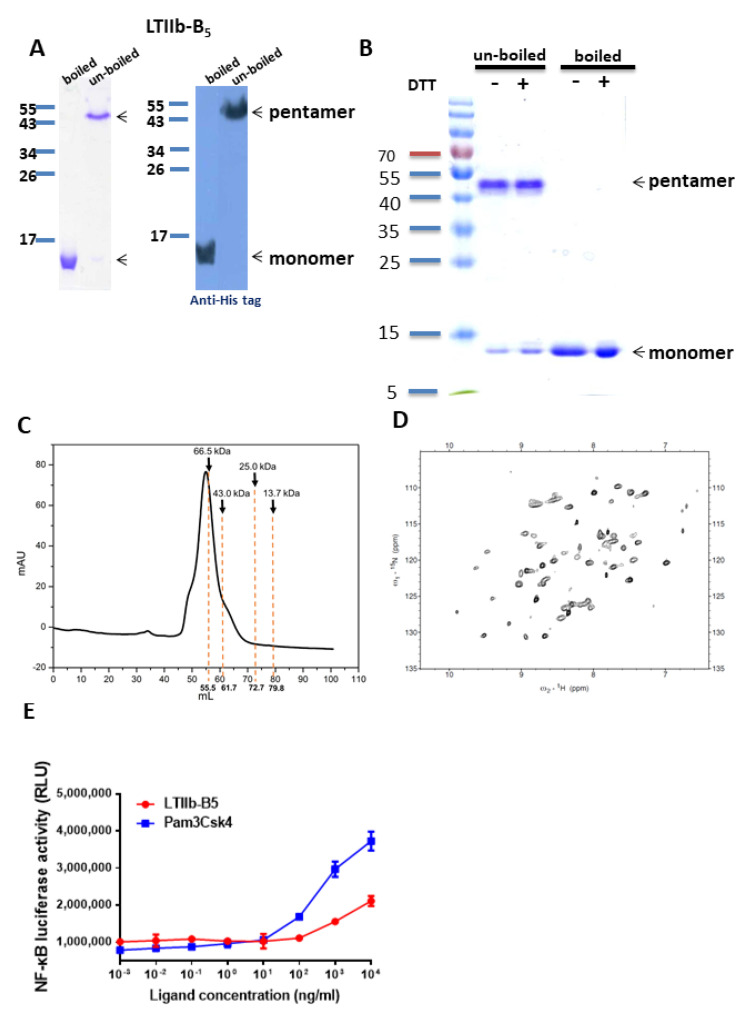
Recombinant LTIIb-B5 protein expression, purification, and characterization (**A**) Left panel, purified LTIIb-B5 protein with Coomassie blue staining; right panel, Western blot. Pentameric LTIIb-B5 is located at ~50 kDa, monomeric LTIIb-B5 at ~12 kDa (denatured). (**B**) Pentameric and monomeric LTIIb-B5 formation under different biochemical conditions. Boiled proteins were heated to 100 °C for 5 min. (**C**) FPLC size-exclusion elution profile for LTIIb-B5 (eluted at 55 mL, corresponding to ~70 kDa). Four marker proteins are Ribonuclease (13.7k), Chymotrypsinogen (25k), Ovalbumin (43k) and Albumin (66.5k). Results indicate the presence of LTIIb-B5 pentamers in solution. (**D**) ^1^H-^15^N HSQC spectrum of LTIIb-B5. Peak dispersion indicates dynamic folded structure. (**E**) TLR2/1 signal functional assay results for LTIIb-B5. HEK 293A cells contained over-expressed hTLR1/hTLR2 receptor and NF-kB-driven luciferase due to transient transfection. Transfected cells were treated with diluted LTIIb-B5 and Pam3CSK4 (1 pg/mL to 10 μg/mL) and held for 5 h at 37 °C. TLR2/1 activity was detected concurrently with luciferase activity.

Recombinant LTIIb-B5 proteins were expressed from *E. coli* and purified using Ni-columns. Residual LPS content of measured by the Limulus amoebocyte assay kit was less than 0.8 EU/ug. Purified proteins, which were analyzed by SDS-PAGE gels with Coomassie blue staining and western blotting (Figure 1A), had monomer sizes of approximately 12 kDa under denaturing conditions (boiling for 5 min). Pentamer formation occurred under native conditions (Figure 1B). The presence or absence of the DTT reducing agent did not change the SDS-PAGE results. When DTT was not present, the denatured LTIIb-B5 remained monomeric, indicating no intermolecular linkages between monomer units (Figure 1B). Previously identified intra-molecular disulfide linkages between Cys10 and Cys81 were maintained during preparation. FPLC gel filtration results indicate that the pentameric form of LTIIb-B5 proteins remained intact (Figure 1C). LTIIb-B5 was eluted at positions corresponding to LTIIb-B5 pentamer molecular size, and structural properties were evaluated using an NMR heteronuclear single quantum coherence (HSQC) spectrometer. Dispersive resonances indicate proper LTIIb-B5 folding (Figure 1D). We also observed that purified LTIIb-B5 proteins were able to trigger the dose-dependent NF-κB activation of TLR-2/1 signaling in HEK 293A cells, thus confirming biological activity (Figure 1E).

### 3.2. LTIIb-B5 Proteins as a Mucosal Adjuvant for HA Protein Immunizations

**Figure 2:**
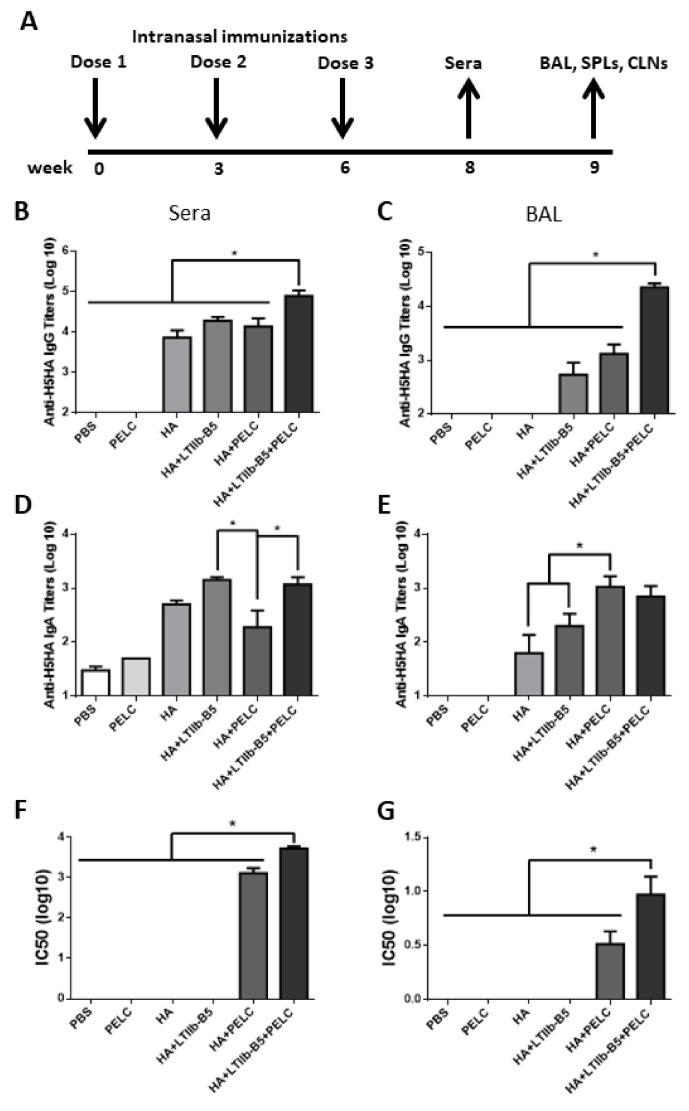
LTIIb-B5 proteins were used as a mucosal adjuvant for HA protein immunizations. (**A**) Three-dose intranasal immunization schedules (five mice per group, n = 5). Groups of BALB/c mice were intranasal immunized with 15 μg HA proteins plus 10% PELC oil-in-water emulsion with or without 1 μg LTIIb-B5 proteins. ELISAs were performed to detect HA-specific IgG titers from (**B**) sera and (**C**) BAL fluids and HA-specific IgA titers from (**D**) sera and (**E**) BAL fluids. Neutralizing antibodies were quantified as reduced luciferase expression levels following H5N1 pseudotyped particle (H5N1pp) transduction in MDCK cells. Corresponding IC50 values were calculated from H5N1pp neutralization curves in (**F**) sera and (**G**) BAL fluids. *, *p* < 0.05.

Groups of BALB/c mice were intranasal immunized with 15 μg HA proteins plus 10% PELC oil-in-water emulsion with or without 1 μg LTIIb-B5 proteins. All groups were immunized three times, at weeks 0, 3 and 6. Sera were collected at week 8, and BAL, SPLs, and CLNs were collected at week 9 (Figure 2A). ELISAs were used to measure HA-specific total IgG and IgA titers in sera and BAL fluid samples. Results indicate that in most immunization groups, the use of LTIIb-B5 adjuvant resulted in significantly higher total IgG and IgA titers in both sera (Figure 2B,D) and BAL fluid (Figure 2C,E). Neutralizing antibodies were determined using H5N1pp assay. IC-50 values indicate that HA proteins formulated in HA + PELC and HA + LTIIb-B5 + PELC adjuvants produced significantly higher neutralizing antibody titers in both sera (Figure 2F) and BAL fluid (Figure 2G), especially in the HA + LTIIb-B5 + PELC adjuvant group. 

**Figure 3:**
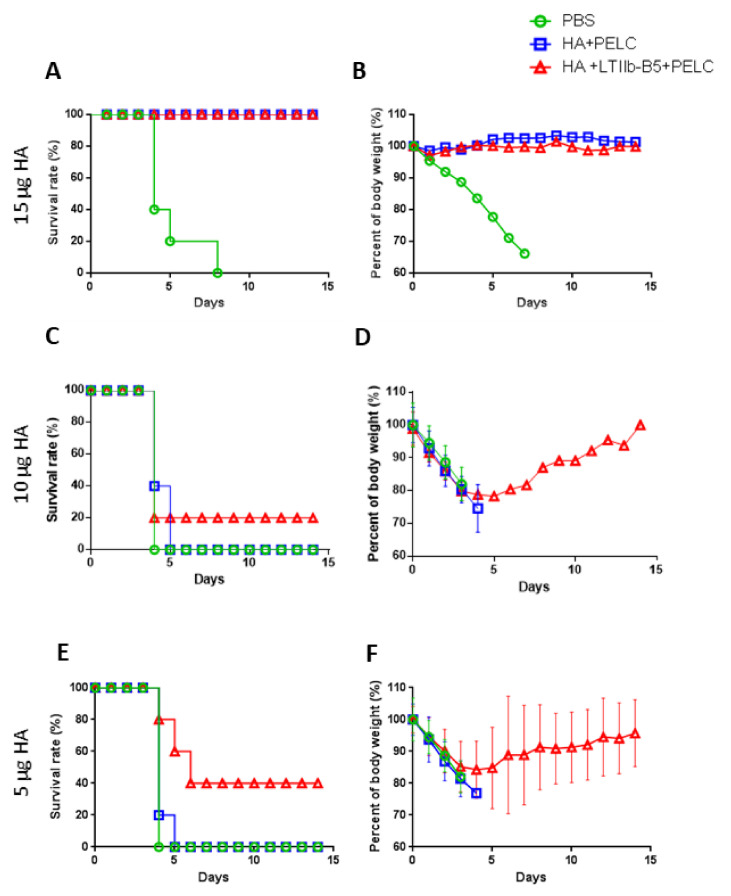
H5N1 viral challenges were performed following intranasal immunization. Mice (five mice per group, n = 5) were immunized with (**A**,**B**) 15 µg (**C**,**D**) 10 µg HA or (**E**,**F**) 5 µg HA. Three weeks after their third immunizations, all mice were given 10 × LD50 of the H5N1 virus (NIBRG-14), also intranasally. Survival rates and body weights were recorded daily for 14 days: blue line, HA + PELC; red line, HA + LTIIb-B5 + PELC; green line, PBS. Body weight loss >25% served as an end-point. Data presented as mean ± S.D.

To further determine protective immunity following HA immunization, we immunized four groups of mice with either 5, 10 or 15 µg of HA antigens plus PELC with or without 1 µg LTIIb-B5 prior to lethal H5N1 virus challenges. A 100% survival rate was observed among mice receiving 15 µg HA antigens both with and without LTIIb-B5 adjuvant (Figure 3A). Furthermore, essentially zero body weight loss was observed in these groups following live virus challenges (Figure 3B). In contrast, a 20% or 40% survival rate was recorded for mice receiving 10 or 5 µg HA antigen immunizations with LTIIb-B5 in a HA + LTIIb-B5 + PELC adjuvant formulation, and 0% survival was observed for non-adjuvant HA + PELC and PBS groups of mice (Figure 3C,E). Full body weight recovery following live virus challenges was only observed in the HA + LTIIb-B5 + PELC immunization group (Figure 3D,F). In sum, the use of LTIIb-B5 as an intranasal HA immunization adjuvant increased protection against influenza virus challenges. 

**Figure 4:**
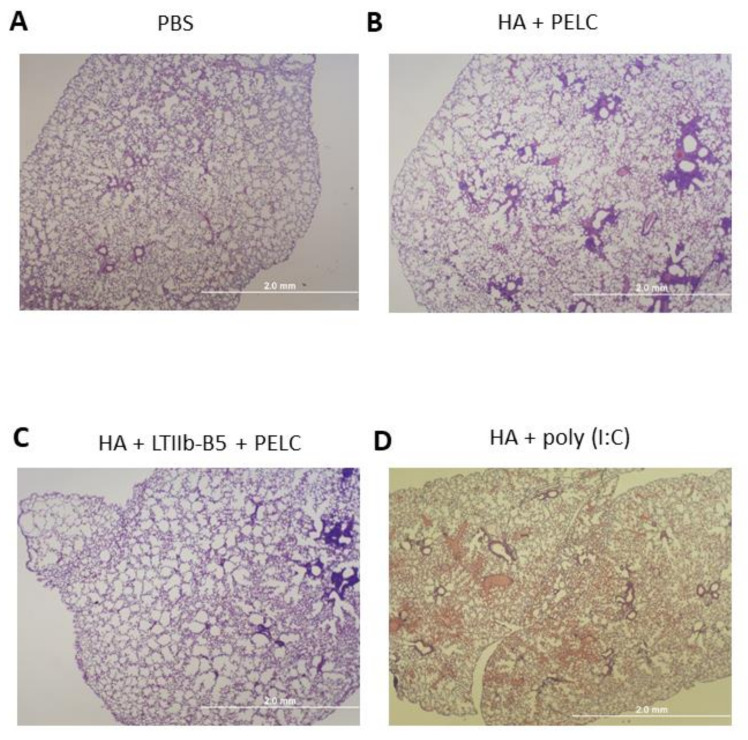
Representative photographs of lung tissue sections (×40 magnification). Mice (five mice per group, n = 5) were intranasally vaccinated with (**A**) PBS, (**B**) 5 μg HA + PELC, (**C**) 5 μg HA + 1 μg LTIIb-B5 + PELC, or (**D**) 5 μg HA +2 μg Poly (I:C). One week after final vaccination, the mice were sacrificed and lung tissue sections collected from mice were stained (H&E) for histopathological examination.

To investigate whether the use of LTIIb-B5 with or without PELC via intranasal route which can cause acute inflammation resulting in sever pneumonia, we further determined the toxicity of the vaccine formulation in the lung using H&E staining. One week after the third dose immunization, the mice were sacrificed and lung tissue sections collected from mice were stained (H&E) for histopathological examination. The microscopic images indicated that like normal ones (Figure 4A), the lung issues of mice received HA + PELC or HA + LTIIb-B5 + PELC (Figure 4B,C) showed no sign of significant histopathological alterations, though they were noticed in a few of the cells where the tissue areas were close to certain bronchi. By contrast, administration of HA + Poly(I:C) caused a plethora of inflammatory cells to infiltrate lung tissues (Figure 4D). Our results showed relatively low inflammation in the absence of cellular infiltration in the lung parenchyma for three-dose intranasal immunizations with the PBS, 5 μg HA + PELC and 5 μg HA + 1 μg LTIIb-B5 + PELC groups as compared to the positive control for 5 μg HA + 2 μg Poly (I:C) (InvivoGen, San Diego, CA, USA) group (Figure 4A–D). Therefore, the adjuvant formation of LTIIb-B5 with or without PELC oil-in-water emulsion resulted in limited cellular inflammation in lung tissues. These results indicate that compared to Poly (I:C), LTIIb-B5 + PELC induced limited pulmonary toxicity in the employed animal model. However, a comprehensive investigation for the safety of LTIIb-B5 + PELC as a mucosal adjuvant for human vaccines are obviously needed.

### 3.3. T Cell Response Detection in SPLs and CLNs

**Figure 5:**
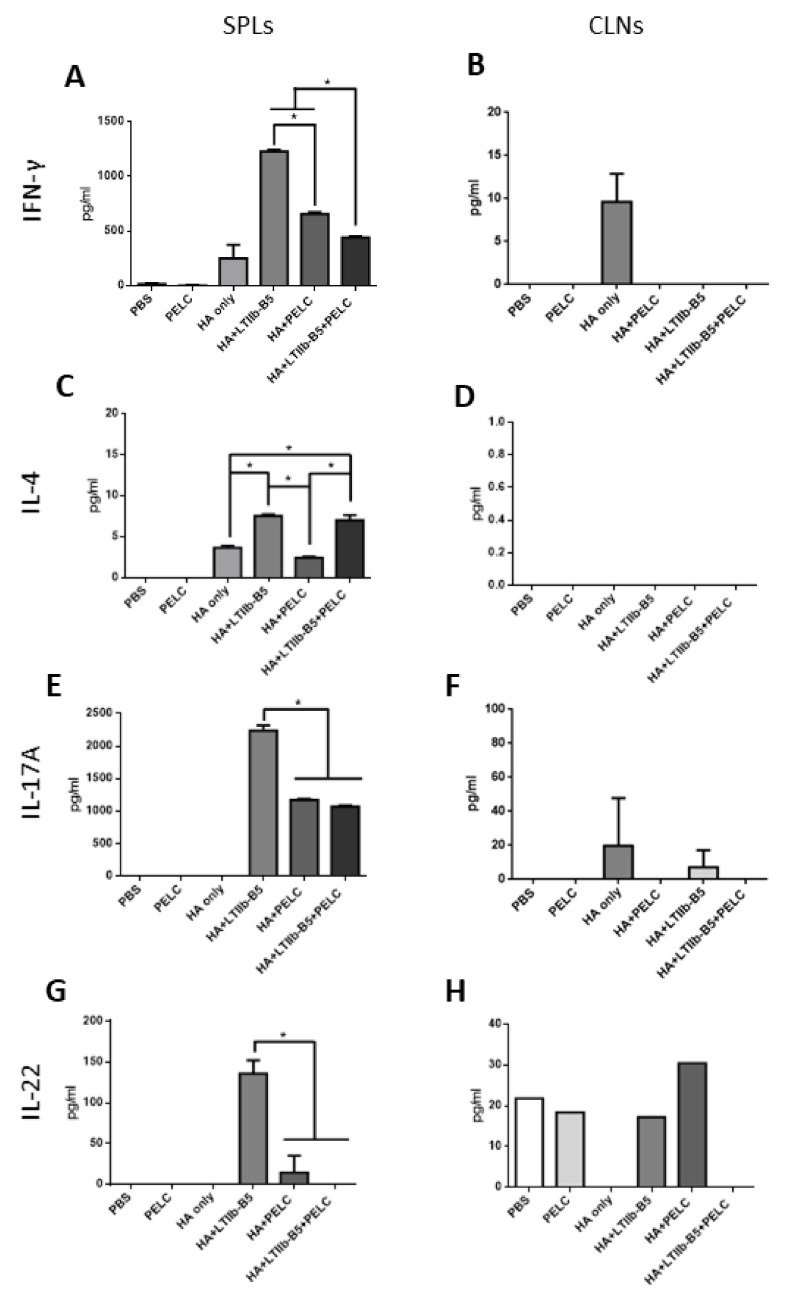
T cell response detection in SPLs and CLNs. Samples of SPLs and CLNs collected from immunized mice (three or four mice per group) were stimulated with 1 μg/mL pooled HA peptides for 72 h. Cytokine titers in cultured supernatants were quantified by ELISAs. (**A**,**B**) IFN-γ cytokines indicate Th1-related responses, (**C**,**D**) IL-4 cytokines indicate Th2-related responses, (**E**,**F**) IL-17A cytokines and (**G**,**H**) IL-22 cytokines indicate Th17-related responses. * *p* < 0.05.

SPLs and CLNs were collected at week 9 and stimulated with pooled HA peptides. ELISAs were performed to determine IFN-γ, IL-4, IL-17A, and IL-22 cytokine levels in culture supernatants. Results indicate that the use of LTIIb-B5 in adjuvant formulation enhanced IFN-γ, IL-4, IL-17A and IL-22 cytokine production in SPLs (Figure 5A,C,E,G). However, these cytokine levels in SPLs were reduced in the presence of PELC oil-in-water formulation (Figure 5A,C,E,G). The levels of IFN-γ, IL-4 and IL-17A cytokine production in CLNs were almost undetectable across all immunized groups (Figure 5B,D,F). The level of IL-22 cytokine production in CLNs for the HA + PELC immunization group was relatively higher than other immunized groups (Figure 5H).

**Figure 6:**
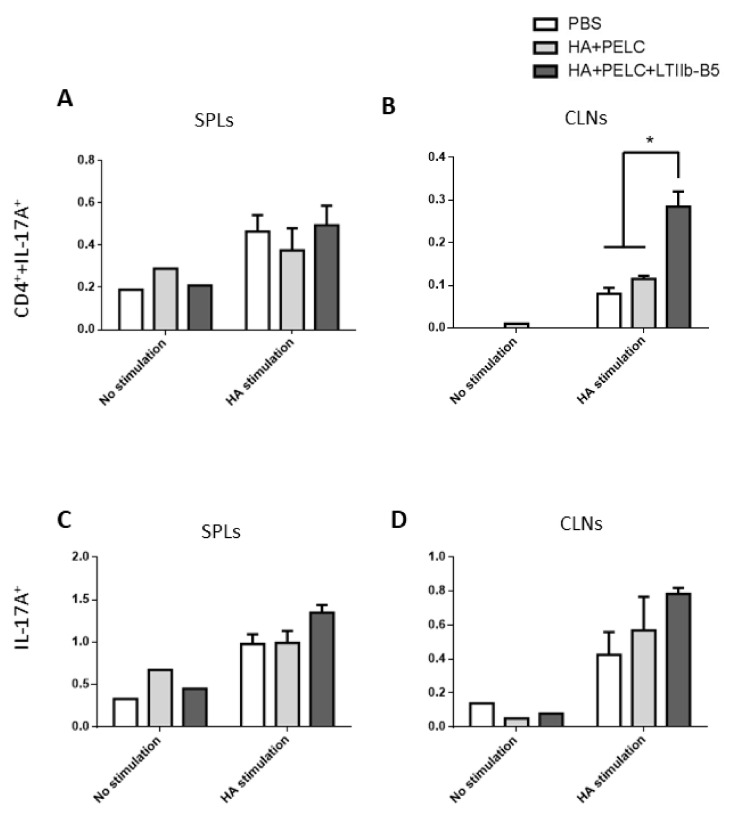
Data from flow analyses performed to determine IL-17A source in SPLs or CLNs. SPLs or CLNs were stimulated with 1 μg/mL pooled HA peptides for 72 h prior to staining with anti-mouse CD4^+^-PE and anti-mouse IL-17A-PerCP-Cy5.5 antibodies. (**A**) The percentage of Th17 cells were calculated from CD4^+^IL-17A^+^ group in SPLs, (**B**) the percentage of Th17 cells were calculated from CD4^+^IL-17A^+^ group in CLNs, (**C**) the percentage of IL-17A-secreating cells were calculated from IL-17A^+^ group in SPLs, and (**D**) the percentage of IL-17A-secreting cells were calculated from IL-17A^+^ group in CLNs. Data were analyzed by one-way ANOVA Holm-Sidak’s multiple comparison tests, * *p* < 0.05.

Flow cytometry data show the increase of CD4^+^IL-17^+^ and IL-17^+^ T cells in SPLs and CLNs with stimulation of pooled HA peptides (Figure 6A–D). In particularly, the percentage of CD4^+^IL-17^+^ CLNs in the HA + PELC + LTIIb-B5 group was significantly higher than the HA + PELC and PBS groups after stimulation of pooled HA peptides (Figure 6B). Thus, the use of LTIIb-B5 adjuvant for intranasal immunizations induced stronger Th17 cellular responses.

### 3.4. Anti-IL-17A Monoclonal Antibody Depletion, IL-17A Knockout Mice and Protective Immunity against H5N1 Challenges

**Figure 7:**
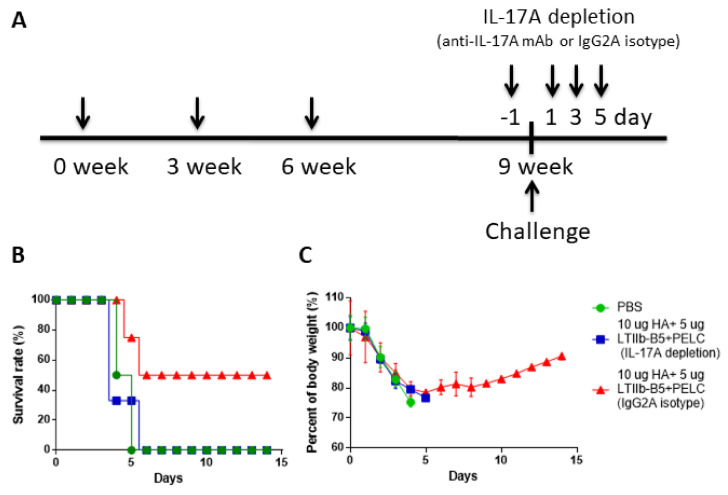
IL-17A depletion data for influenza virus protective immunity. (**A**) Intranasal immunization schedules. Mice (four mice per group, n = 4) were immunized with 10 µg HA plus 5 µg LTIIb-B5. Three weeks following their third immunizations, mice were intranasal challenged with 10 × LD50 of the H5N1 virus (NIBRG-14). (**B**) Survival rates and (**C**) body weights were recorded daily for 14 days. Blue line, immunized mice treated with IL-17A monoclonal antibodies; red line, mice treated with the IgG2A isotype Abs; green line, mice treated with PBS only (no Abs). Body weight loss >25% was used as an end-point. Loss data are presented as mean ± S.D. Identical procedures were followed with another group of mice. Collected SPLs and CLNs were stimulated with 1 μg/mL pooled HA peptides for 72 h.

To determine whether a link exists between increased Th17 cellular response and protective immunity against live virus challenges, we injected anti-IL-17A monoclonal antibodies and IgG2A isotype control antibodies into BALB/c mice in the HA + PELC + LTIIb-B5 group 1 day before and 1, 3 and 5 days following viral challenges (Figure 7A). To increase the survival rate in the immunized mice, we changed the amount of LTIIb-B5 adjuvant (from 1 μg to 5 μg) at 10 μg HA per dose for IL-17 depletion experiments. Results indicate that IL-17A depletion following anti-IL17 mAb injections triggered a decline in survival from 50% (control group) to 0% (Figure 7B). No body weight recovery was observed for the HA + LTIIb-B5 immunization group following injections with anti-IL-17A mAb, as compared to the control group using anti-IgG2A mAb (Figure 7C). These results suggest that the protective immunity observed in HA + PELC + LTIIb-B5-immunized mice was linked to Th17 cellular response.

**Figure 8:**
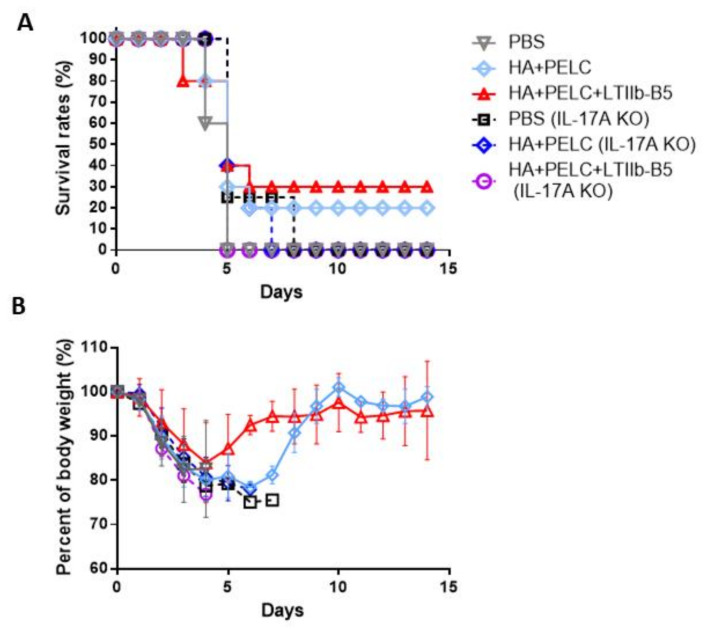
LTIIb-B5-induced protection was countervailed in IL-17A knockout mice. Immunized mice (four, five or ten mice per group) treated with 10 µg HA plus 5 µg LTIIb-B5 were intranasal challenged with 10 × LD50 of the H5N1 virus (NIBRG-14) 3 weeks following their third immunizations. (**A**) Survival rates and (**B**) body weights of mice immunized with PBS; gray line, C57BL/6 wild-type mice; black line, PBS in C57BL/6 IL-17A KO mice; light blue line, HA + PELC in C57BL/6 wild-type mice; deep blue line, HA + PELC in C57BL/6 IL-17A KO mice; red line, HA + LTIIb-B5 + PELC in C57BL/6 wild-type mice; and purple line, HA + LTIIb-B5 + PELC in C57BL/6 IL-17A KO mice. Data were recorded daily for 14 days. Body weight loss >25% was used as an end-point. Data are presented as mean ± S.D.

We followed the same immunization procedures with IL-17A knockout (IL-17A KO) and wt C57BL/6 mice. For wild-type C57BL/6 mice, those in the HA + PELC and HA + PELC + LTIIb-B5 groups following viral challenges exhibited 20% and 30% survival rates, respectively. However, the same immunization regimens resulted in 0% survival rates for IL-17A KO mice (Figure 8A). Body weight recovery was only observed for wild-type C57BL/6 but not IL-17A KO mice in the HA + PELC and HA + PELC + LTIIb-B5 groups (Figure 8B). Also, an early weight recovery was observed for the HA + PELC + LTIIb-B5 group compared to the HA + PELC group (Figure 8B). Combined, these results suggest that the LTIIb-B5 adjuvant improved the protective immunity of mucosal HA vaccines via enhanced TH17 cellular immunity.

## 4. Discussion

Recombinant LTIIb-B5 proteins have been shown to form native pentameric structures from monomers. According to our NMR evaluation data, recombinant LTIIb-B5 expressed a well-folded property due to the dispersive distribution of HSQC spectrum resonances. Only one set of resonances corresponding to pentamer formation was identified, and no LTIIb-B5 monomer or other oligomeric formations were detected, indicating stability in LTIIb-B5 pentamer structure. Monomers in the same pentamer complex expressed the same conformations. TLR-2/1 activity was also displayed after boiling (data not shown). Further investigation is needed to clarify molecular interactions between TLR2/1 and pentamer complexes.

In the current study, the adjuvant effects of LTIIb-B5 show the increased IgG and IgA titers in sera and BALs, and higher numbers of IFN-γ and IL-17A secreting T cells in SPLs (Figure 5 and Figure 6). We incorporated the use of PELC oil-in-water emulsion with HA and LTIIb-B5 to facilitate the uptakes of antigen (HA) and adjuvant (LTIIb-B5) molecules targeting to the same antigen-presenting cells and their pattern recognition receptors for better antigen presentation regardless PELC has adjuvant properties. The combination of antigen (HA) and adjuvant (LTIIb-B5) proteins into single droplets of PELC oil-in-water micelles can be physically linked through the hydrophilic arms of PELC oil-in-water micelles attaching the soluble proteins of HA and LTIIb-B5 molecules. The combination of HA + LTIIb-B5 + PELC was found to achieve highest titers of neutralizing antibodies in sera and BAL fluids.

Both *E. coli* heat labile enterotoxins and cholera toxins are well-studied mucosal adjuvants inducing secretory IgA [22]. Increased secretory IgA production following use of the LTIIb-B5 adjuvant may be due to an increase in pro-inflammatory Th17 cells [23,24]. However, three-dose immunizations are required to induce significant levels in sera and mucosal fluids for anti-H5N1 immune responses as we previously reported for intranasal immunizations with HA and recombinant flagellin in the PELC oil-in-water emulsions [25]. A similar dosage of HA for three-dose intranasal immunization was documented for chitosan nanoparticles (15 μg HA per dose) [26,27] or co-delivery with a mast cell activator protein (9 μg HA per dose) [28]. In general, mucosal delivery generally needs higher antigen content and multiple booster doses to induce effective immune responses. Additionally, the essential question for the long-term anti-HA memory responses elicited by use of LTIIb-B5 and PELC adjuvants for mucosal immunization still require further investigation.

To investigate T cell responses elicited by the LTIIb-B5 adjuvant, we analyzed IFN-γ, IL-4, IL-17A and IL-22 cytokine levels in SPLs and CLNs stimulated with pooled HA peptides. Significant increases in IL-17A and IL-22 cytokine production in mice immunized with HA+LTIIb-B5 indicate that the LTIIb-B5 adjuvant elicited more potent Th17 cell responses that subsequently contributed to protection against H5N1 virus challenges. A likely explanation is that LTIIb-B5 functions via TLR-dependent pathways to modulate IL-17A responses in hosts [29]. Several studies have reported TLR-2-dependent signaling pathways for Th17 cell polarization via either TLR-2 ligands or induction by periodontal pathogens [30,31]; Th17 responses in nasal-associated lymphoid tissues were induced by group A streptococcus declined in TLR-2^−/−^ mice [32]. Results from our challenge experiments by anti-IL17 mAb injections in BALB/c mice suggest that the anti-H5N1 protective immunity was linked to Th17 cellular response (Figure 7). We followed the same immunization procedures with IL-17A KO mice (C57BL/6) to show the losses of survival and body weight recovery (Figure 8). The protective role of Th17 response against H1N1 influenza virus infections has been previously documented [33,34]. In contrast, Th17 response mediated by increases in tissue myeloperoxidase due to elevated numbers of lung neutrophils was reported for increased morbidity [35]. Intranasal immunization with a split H3N2 vaccine plus a TLR-4 agonist induced polyfunctional Th17 cellular responses to exacerbate inflammation and increase weight loss and morbidity during early influenza infections [36]. It is also likely that Th17 mediated protective responses may involve the recruitment of neutrophils, release of anti-microbial peptides and IL-17-driven Th1 immunity [37]. Additionally, IL-17A can induce pro-inflammatory cytokines that communicate with other forms of adaptive immune response and that recruit neutrophils to inflammatory sites [38]. Innate B-1a cells can differentiate via IL-17A modulation into vigorous IgM-producing cells responding to influenza infections [39]. Th17 cell localization and macrophage activation via CXCL13 expression can be triggered simultaneously by mucosal *Mycobacterium tuberculosis* peptide vaccination with the use of LTIIb adjuvant [40]. Details on the Th17 cellular response mechanisms that confer anti-H5N1 protective immunity have yet to be determined.

*E. coli* heat labile enterotoxin contains a single enzymatically active A subunit and a pentameric B subunit. The B pentamer subunit mediates binding of the holotoxin to ganglioside receptors, which are a family of cell surface glycolipids found ubiquitously on mammalian cells [41]. Different heat labile enterotoxins (LT-I, LTIIa, LTIIb, LTIIc) bind to unique gangliosides or with varying affinity to members of a subset of gangliosides. While evidence of LTIIb being an adjuvant candidate for mucosa immunization is accumulating from independent studies including our current one, safety remains a major concern. Earlier experience of using intranasal influenza vaccine composed of influenza antigens with *E. coli* derived LT-I adjuvant, resulted in a total withdrawal of the vaccine due to a strong association of the vaccine and Bell’s palsy [41]. The mechanism underlying Bell’s palsy after intranasal vaccination remained not totally understood, but the role of LT-I adjuvants cannot be ignored. Clinical trials using a genetically detoxified mutant A subunit of LT-I (LTK63) along or as a vaccine adjuvant also resulted in transient facial palsy [42]. Although LTIIb-B5 shares very limited primary amino acid homology or biological properties with LT-I, a comprehensive investigation of the safety of LTIIb-B5 as an adjuvant for human vaccines are obviously needed.

## 5. Conclusions

We showed evidence that LTIIb-B5 protein, when used with intranasal HA protein as an adjuvant, induced higher HA-specific IgG, IgA, and neutralizing antibodies, more Th17 cells in SPLs and CLNs, and greater protection against live virus challenges. Our results also indicate a link between improved protection against H5N1 live virus challenges and increased Th17 response due to the use of LTIIb-B5 mucosal adjuvant with HA subunit proteins. These findings may provide useful information for mucosal H5N1 subunit vaccine development.

## Data Availability

All data generated during this study are included in this published article.
